# Impact of Frailty on Surgical Outcomes in Nonacute Subdural Hematomas: A Nationwide Analysis of 251,597 Patients over 20 Years

**DOI:** 10.3390/jcm14093176

**Published:** 2025-05-04

**Authors:** Avi A. Gajjar, Nathan Ramachandran, Tarun Prabhala, John Y. Chen, Amanda Custozzo, Alexandra R. Paul

**Affiliations:** Department of Neurosurgery, Albany Medical Center, Albany, NY 12208, USA

**Keywords:** frailty, nonacute subdural hematoma, cSDH, craniotomy, chronic subdural, Charlson comorbidity index, modified frailty index

## Abstract

**Background/Objectives**: Nonacute subdural hematomas (naSDHs) are a prevalent intracranial pathology, particularly in older people, due to increased brain atrophy, fall risk, and anticoagulant use. This study examines the impact of frailty on the surgical outcomes of craniotomy for naSDH over 20 years. **Methods**: Data from the Nationwide Inpatient Sample (NIS) from 2000 to 2021 were analyzed, including 251,597 patients who underwent cranial decompression for naSDH. Patients were selected using specific ICD codes. Frailty was calculated using the modified frailty index (mFI-5 and mFI-11) and the Charlson Comorbidity Index (CCI). Outcomes were compared using descriptive statistics and multivariable regression models. **Results**: 251,597 patients underwent craniotomy, with a mean age of 69.2 years. The cohort exhibited significant comorbid conditions, reflected in a mean Charlson Comorbidity Index (CCI) of 3.8, and a high frailty prevalence, with 23.49% of patients classified as frail and 20.14% as severely frail. The CCI demonstrated the highest predictive value for adverse outcomes, with an area under the curve (AUC) of 0.6346 for mortality and 0.6804 for complications. Frailty indices (mFI-5 and mFI-11) were also strongly associated with increased mortality (*p* < 0.001), complications (*p* < 0.001), and extended length of stay (*p* < 0.001). Age was not a significant predictor of outcomes. **Conclusions**: This study highlights the moderate impact of frailty on surgical outcomes for naSDH.

## 1. Introduction

Nonacute subdural hematomas (naSDHs) are a prevalent intracranial pathology, particularly in older people, due to increased brain atrophy, fall risk, and anticoagulant use [1,2]. The incidence of naSDHs currently ranges from 1.7 to 20.6 per 100,000 individuals, increasing with age and reaching a peak among those 70 years and older [3,4]. Given that this population sector is projected to increase by 300% in 2050, the anticipated rise in naSDH cases could significantly impact neurosurgical practices and the healthcare system [2].

Surgical evacuation is the mainstay of treatment for naSDH. Open craniotomy allows for maximal access and the visualization of the subdural space, enabling the comprehensive excision of membranous debris and the evacuation of chronic subdural blood products [3,5]. However, this more invasive technique is associated with longer operating times, higher volumes of blood loss, more postoperative complications, and longer recovery periods, particularly for frail patients [3,6,7]. Craniectomy involves delaying the replacement of the excised skull and is indicated for patients at higher risk of morbidity from brain swelling. Craniostomy, also known as burr hole trephination, is considered the least invasive and can be conducted at the bedside, although it provides the least visualization.

Several recent studies have emphasized the clinical value of frailty assessment in cSDH management. Shimizu et al. examined 211 elderly patients undergoing surgery for cSDH [8]. They found that frailty was strongly associated with worse functional outcomes, with frail patients significantly less likely to be discharged home and more likely to have poor modified Rankin Scale scores at three months. Although multivariate analysis did not retain frailty as an independent predictor, adding frailty to prognostic models significantly improved predictive accuracy, indicating that frailty reflects important physiological reserve beyond age or comorbidity alone [8].

This association between frailty and poor outcomes has been corroborated in larger retrospective studies. Sastry et al. analyzed data from 1647 patients undergoing craniotomy for atraumatic cSDH, which demonstrated that increasing frailty, as measured by the mFI-5, was independently associated with higher odds of major complications, non-home discharge, and 30-day mortality [9]. Their findings suggest that frailty stratification may be critical for surgical risk assessment and discharge planning in patients with chronic subdural collections [9]. Kesserwan et al. similarly demonstrated that frailty strongly predicted discharge dependence and poor functional improvement after twist-drill craniostomy. The Clinical Frailty Scale outperformed the mFI in predicting both independence and improvement, highlighting the relevance of multidimensional frailty assessment [10].

The broader epidemiological context further underscores the importance of frailty in naSDH care. A retrospective cohort analysis of cSDH paired with general population mortality rate found that cSDH patients had significantly higher long-term mortality than age-matched controls, with increased deaths from cardiovascular causes and falls, and with functional impairments such as poor self-care and dependence correlating with mortality risk [11]. Their data demonstrated that frailty of cSDH may be a more clinically meaningful prognostic factor than chronological age alone in surgical decision-making for naSDH.

The predictive value of various indices, including the 5-factor modified frailty index (mFI-5), 11-factor modified frailty index (mFI-11), and the Charlson Comorbidity Index (CCI), can provide a more nuanced understanding of outcomes than age alone [12]. Frailty influences surgical outcomes, making it essential to tailor prognosis based on individual patient profiles. Given the prevalence of naSDHs in older people, exploring the potential of frailty in guiding patient-oriented decisions on surgical selection is helpful. While prior investigations into frailty and chronic subdural hematoma have been limited to single-center, retrospective cohorts, large-scale, population-based analyses remain lacking. This study addresses that gap by evaluating national-level outcomes over 20 years. We aim to compare the national outcomes of craniotomy for naSDH, focusing on frailty as a predictive factor.

## 2. Materials and Methods

This research employed the Nationwide Inpatient Sample (NIS), the largest comprehensive inpatient care database in the United States [13]. The NIS is a 20% stratified sample of hospital inpatient admissions nationwide, encompassing around 97% of the U.S. population. Approximately 7 million unweighted inpatient records are compiled annually in the database, detailing diagnostic and procedural information through the International Classification of Diseases, Tenth Revision, Clinical Modification, and the Procedure Coding System (ICD-10-PCS). The NIS is a valuable resource for examining nationwide trends in inpatient care and outcomes. The database includes data on patient demographics, diagnoses, procedures, length of stay, discharge status, hospital characteristics, and more. It is useful to do a comprehensive analysis of various healthcare outcomes and trends across the USA, due to its significant population coverage. No Institutional Review Board (IRB) approval was necessary for this study, as it utilized de-identified public data and is thus IRB-exempt. The study was developed and reported according to the guidelines for the STROBE (Strengthening the Reporting of Observational Studies in Epidemiology).

Patients were selected based on specific ICD codes. The primary or secondary diagnosis of nontraumatic chronic subdural hemorrhage was identified using ICD-9-CM code 432.1 and ICD-10-CM code I62.03. In cases where the attending physician specified it, the code I62.00 for nontraumatic subdural hemorrhage, unspecified, was also used. As clinical granularity is limited in administrative datasets, we used previously validated coding approaches to optimize case identification [14,15]. Thus, we included all patients who underwent surgical evacuation for the treatment of non-acute subdural hematoma. Complications were categorized into several domains: cardiac, pulmonary, thromboembolic, infectious (central nervous system and wound-related), renal/genitourinary, gastrointestinal, deep vein thrombosis, and sepsis. The criteria for these complications were based on previously established studies [16,17].

Frailty was assessed using the modified frailty indices (mFI-5 and mFI-11), which are validated tools used to quantify the frailty status of patients. The mFI-5 includes five variables: functional status, diabetes, chronic obstructive pulmonary disease (COPD), congestive heart failure, and hypertension requiring medication. Each comorbidity is assigned a value of 1, with total scores ranging from 0 to 5. The mFI-11 expands on this by including additional variables such as history of myocardial infarction, peripheral vascular disease, cerebrovascular accident, dementia, and connective tissue disease/rheumatologic disease, resulting in scores ranging from 0 to 11. Higher scores indicate greater frailty and are associated with an increased risk of adverse postoperative outcomes [18,19].

The Charlson Comorbidity Index (CCI) was used to assess the burden of comorbidities. The CCI assigns different weights to various comorbid conditions based on severity, with a possible score range from 0 to 28. Conditions included in the CCI are myocardial infarction, congestive heart failure, peripheral vascular disease, cerebrovascular disease, dementia, chronic pulmonary disease, connective tissue disease, peptic ulcer disease, liver disease, diabetes, hemiplegia, moderate or severe renal disease, any malignancy, lymphoma, leukemia, and acquired immune deficiency syndrome [20]. For each condition, a score is assigned, and these scores are summed to produce a total CCI score. This score estimates the mortality risk or severe outcomes associated with the patient’s comorbidities. Our study calculated CCI using ICD-9-CM and ICD-10-CM codes, with a methodology consistent with prior studies [13]. Craniotomy procedures were identified using a validated set of ICD-9 and ICD-10 procedural codes consistent with the method outlined by Tang et al. in the Neurosurgery Primer for national database research [20].

The statistical analysis adhered to HCUP guidelines, utilizing trend weights to adjust for the NIS sampling design and generating representative national estimates. Descriptive statistics were employed to assess baseline demographic and clinical characteristics, complications, and outcomes across different patient subgroups. Dichotomous variables were examined using Pearson’s chi-square test, and continuous variables were analyzed through generalized linear regression models incorporating a gamma distribution and logistic link function. Multivariable (MV) regression models were adjusted for confounders, including age, sex, race, insurance status, income quartile, hospital ownership, teaching status, and bed size. Binary outcomes were evaluated using odds ratios (ORs) with 95% confidence intervals, indicating the association’s strength. For example, an OR of 1.05 indicates a 5% higher likelihood of the outcome occurring when the specified factor is present. We used gamma regression with a log-link function (GLM) to model hospital cost, as it appropriately accounts for the right-skewed distribution of cost data and provides interpretable estimates of multiplicative effects, with significance evaluated using *p*-values. Receiver operating characteristic (ROC) curves were made for each frailty score to assess their predictive value for discharge disposition. The high frailty threshold for mFI-5, mFI-11, and CCI was defined at the point on the ROC curve where Youden’s index reached its maximum, with a threshold score set at ≥2 for all indices. Previous research has corroborated the link between preoperative frailty and hospital discharge outcomes in neurosurgery, reinforcing the use of discharge status as a functional proxy [21,22]. Statistical significance was set at an alpha = <0.05, and all analyses were conducted using STATA/SE Version 18.0 (StataCorp, College Park, TX, USA).

## 3. Results

### 3.1. Patient Demographics and Characteristics

A total of 251,597 patients who underwent craniotomy were identified from the NIS database. The mean age across all patients was 69.2 years (SD: 14.7), with the majority aged 65–79 years (40.5%) (Table 1). This age group remained the majority from 2000 to 2020, comprising 40.5% of all craniotomy patients in both years. Males constituted 66.2% of the overall cohort (Table 1). Medicare was the primary payer for most patients (64.8%). Geographically, the South accounted for the highest percentage of hospitalizations (38.6%), and most were treated in urban teaching hospitals (71.2%).

### 3.2. Comorbidities and Healthcare Utilization

The CCI indicated a significant burden of comorbid conditions within the craniotomy cohort, with a mean CCI of 3.8 (SD: 2.0). The median length of stay was 8.9 days (SD: 8.5), with total charges averaging USD 117,090 (SD: USD 115,136) and an estimated cost of care at USD 28,492 (SD: USD 24,979). Black patients had the most extended average length of stay of 10.4 days (SD: 10.3), while Hispanic patients had the highest total charges at USD 128,812 (SD: USD 152,552). Complications occurred most frequently in frail (27.6%) and severely frail patients (34.9%) (Table 2). Non-home discharge occurred in 68.4% and 76.2% of frail and severely frail craniotomy patients, respectively. The rate of complications increased from 17.4% in 2000 to 30.1% in 2020. Black and Native American patients accounted for the most significant proportion of complications, with 31.0% and 33.1%, respectively. Frailty, as measured by the modified frailty index (mFI-5 and mFI-11), varied significantly within the cohort, with 25.98% of patients classified as being robust, 30.38% pre-frail, 23.49% frail, and 20.14% severely frail.

### 3.3. ROC Curve Predictive Value of Frailty Indices

**Figure 1:**
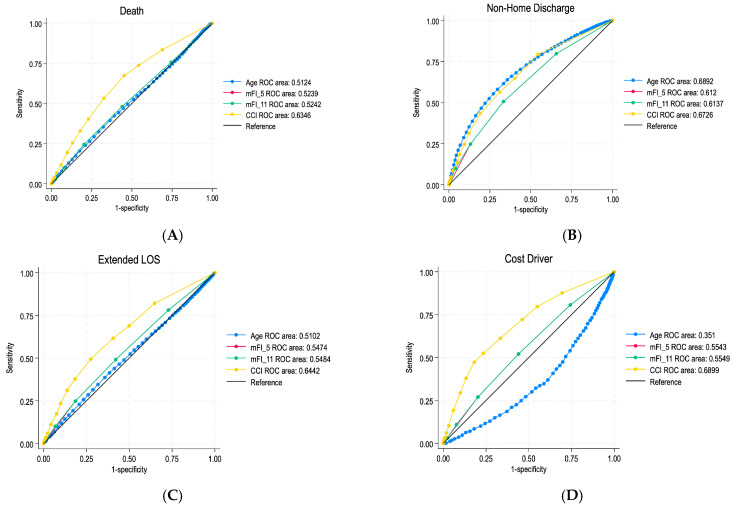
ROC curves for (**A**) death, (**B**) non-home discharge, (**C**) extended length of stay (LOS), and (**D**) cost drivers.

The CCI demonstrated the highest predictive value for multiple outcomes across all craniotomy patients. For the cohort, the CCI had an area under the curve (AUC) of 0.6346 for mortality, outperforming mFI-5 (0.5239), mFI-11 (0.5242), and age (0.5124) (Figure 1). For non-home discharge, all indices performed better, with the CCI leading (AUC 0.6726) compared to mFI-5 (AUC 0.612), mFI-11 (AUC 0.6137), and age (AUC 0.6892). The CCI also led in predicting complications (AUC 0.6804) and extended length of stay (AUC 0.6442). Regarding cost drivers, the CCI was the most reliable predictor (AUC 0.6899). The frailty indices (mFI-5: AUC 0.5543, mFI-11: AUC 0.5549) followed, while age (AUC 0.351) exhibited an inverse association with cost, reflecting its inferior predictive value.

### 3.4. Multivariable Analysis of Predictive Value of Frailty Indices

Multivariable analysis for all craniotomy patients revealed that mFI-5, mFI-11, and CCI were significant predictors across various outcomes (Table 3) (Supplementary Tables S1–S3). The mFI-5 was strongly associated with increased mortality (OR: 1.091, 95% CI: 1.048–1.135, *p* < 0.001), non-home discharge (OR: 1.359, 95% CI: 1.326–1.394, *p* < 0.001), complications (OR: 1.355, 95% CI: 1.323–1.389, *p* < 0.001), and extended length of stay (OR: 1.147, 95% CI: 1.120–1.175, *p* < 0.001). For cost, the gamma log-link beta coefficient (2645, *p* < 0.001) suggests an exponential association, with higher frailty indices linked to markedly increased costs. The mFI-11 produced similar predictive results. The CCI also exhibited strong associations with increased mortality (OR: 1.151, 95% CI: 1.140–1.163, *p* < 0.001), non-home discharge (OR: 1.230, 95% CI: 1.219–1.242, *p* < 0.001), complications (OR: 1.227, 95% CI: 1.218–1.237, *p* < 0.001), and extended length of stay (OR: 1.182, 95% CI: 1.173–1.190, *p* < 0.001). For cost, the gamma log-link beta coefficient (OR: 2494, *p* < 0.001) highlights a robust exponential relationship, underscoring the significant economic burden associated with higher comorbidity scores. Age was only a significant predictor for non-home discharge (OR: 1.039, CI: 1.037–1.041, *p* < 0.001) (Supplementary Table S4).

## 4. Discussion

This study analyzes the inpatient outcomes of surgical evacuation for treating naSDHs, focusing on patient demographics, complication rates, and costs over 20 years. Surgical evacuation, which allows for the comprehensive excision of membranes and evacuation of hematoma, was associated with a high complication rate, as evidenced by the 25% complication rate and 8% inpatient mortality.

On the other hand, our multivariable analysis revealed that age was not a significant predictor of most outcomes in the cohort, challenging the traditional reliance on age-based criteria for surgical decision-making. This supports the growing evidence that frailty, rather than age alone, is a more accurate predictor of surgical risk [23]. Prioritizing frailty over age in patient assessments can enable clinicians to identify high-risk patients better and tailor surgical and perioperative strategies accordingly [24,25]. Our findings are consistent with broader research identifying frailty as a critical factor in surgical risk stratification, with strong associations observed between frailty and postoperative mortality and complications [24,26,27].

The CCI demonstrated superior predictive performance across multiple outcomes, highlighting the importance of evaluating comorbid conditions holistically in determining surgical approach and candidacy [28]. However, frailty indices such as the mFI-5 and mFI-11 also showed significant predictive value, suggesting that frailty metrics can capture aspects of patient health not fully accounted for by the traditional age measure [29]. However, most ROC curves demonstrated an AUC < 0.70, indicating improved performance over age alone but limited overall discriminative ability. While frailty indices such as mFI-5, mFI-11, and the CCI [24,25] outperformed age in predicting mortality, complications, and discharge disposition, the overall discriminative ability of these models remained modest, with AUC values below 0.70 for most outcomes. This suggests that although frailty offers clinically valuable insight, it should not be viewed as a standalone tool for prognostication. Instead, frailty should be integrated into a broader risk assessment framework, including functional status, imaging findings, and perioperative factors.

Quantitative frailty indices support more precise surgical planning and perioperative risk stratification [30]. Surgeons can provide clearer expectations of risks and outcomes by discussing frailty metrics with patients and families, facilitating shared decision-making, and allowing for shared decision-making [31]. For patients with a higher frailty score, a surgeon may wish to consider less invasive procedures such as bedside Subdural Evacuating Port System^TM^ (Medtronic, North Haven, CT, USA) placement or possibly middle meningeal artery embolization. Other patient-specific strategies have been suggested to mitigate the adverse effects of frailty, such as oral protein supplementation and nutrition therapy for patients suffering from malnutrition or perioperative physical exercise regimens [32]. Moreover, even without these additional resources, simply raising awareness of frailty and making minor revisions to hospital logistics has been found to result in a decrease in mortality in naSDH patients [11,33,34]. 

Several prior studies have associated frailty and outcomes in patients with chronic subdural hematomas, primarily in single-center, retrospective cohorts with limited sample sizes [8,9,10]. While these studies demonstrated that frailty predicts poor discharge status and increased complications, they were limited in generalizability. Our study builds on this existing work by providing a national-level analysis encompassing over 250,000 patients treated over two decades. To our knowledge, this is the largest cohort assessing frailty using multiple indices in the context of naSDH surgery, thereby addressing a critical gap in the literature related to large-scale outcome prediction and risk stratification.

However, several limitations should be considered. The retrospective design and reliance on the NIS leads to inherent selection bias and coding errors [20]. The NIS does not contain clinical nuances, such as specific hematoma size, functional status, and more. The exclusion of patients who did not undergo surgery also potentially omits those who were too sick to receive treatment. Due to the cross-sectional nature of the NIS database, we could not account for repeat admissions or distinguish initial craniotomies from reoperations. As such, repeat craniotomies may be included in the cohort, which could influence complication or outcome rates. 

Furthermore, the absence of long-term outcomes at 3, 6, and 9 months limits our understanding of the full impact of the surgical interventions. Additionally, studies should explore using discharge disposition and short-term functional status as surrogates for long-term neurological and quality-of-life outcomes. These measures may offer pragmatic endpoints in administrative datasets where long-term follow-up is unavailable. 

## 5. Conclusions

In conclusion, our comprehensive 20-year analysis offers important insights into outcomes following open craniotomy for nonacute subdural hematomas. These findings demonstrate that patient frailty can determine postoperative outcomes for cSDH patients. This demonstrates the greater limitations of relying solely on age, rather than frailty, when selecting cSDH drainage candidates. Incorporating frailty into clinical decision-making allows for more accurate risk stratification and supports individualized treatment planning, particularly in an aging population. Tailored surgical strategies and patient-centered discussions should reflect physiological reserve rather than age alone. Future prospective studies are needed to validate these population level observations and optimize surgical management of nonacute subdural hematomas.

## Figures and Tables

**Table 1:** Demographics and patient characteristics.

Label	naSDH Patients
**Total Number of Patients**	251,597 (100.0%)
**Mean Age**	69.171 (14.666)
**Age Group**	
18–29	4040 (1.6%)
30–49	21,789 (8.7%)
50–64	56,211 (22.3%)
65–79	101,968 (40.5%)
80+	67,589 (26.9%)
**Sex**	
Male	166,334 (66.2%)
Female	85,027 (33.8%)
**Race**	
White	143,733 (67.5%)
Black	30,142 (14.2%)
Hispanic	20,596 (9.7%)
Asian	10,020 (4.7%)
Native American	1255 (0.6%)
Other	7078 (3.3%)
**Payer**	
Medicare	162,667 (64.8%)
Medicaid	17,623 (7.0%)
Private	54,665 (21.8%)
Self-Pay	8048 (3.2%)
No Charge	694 (0.3%)
Other	7431 (3.0%)
**Region of Hospital**	
Northeast	43,633 (17.3%)
Midwest	51,733 (20.6%)
South	97,169 (38.6%)
West	59,062 (23.5%)
**Location/Teaching Status of Hospital**	
Rural	7163 (2.9%)
Urban Non-teaching	64,891 (25.9%)
Urban Teaching	178,535 (71.2%)
**Charlson Comorbidity Index**	3.842 (3.019)
**Hospital Bed Size**	
Small	14,779 (5.9%)
Medium	51,769 (20.7%)
Large	184,040 (73.4%)
**Median Household Income Quartile**	
1	58,528 (23.8%)
2	60,256 (24.6%)
3	60,845 (24.8%)
4	65,800 (26.8%)
Length of Stay	8.945 (8.481)
Total Charges	92,443.182 (112,750.623)
Estimated Cost of Care	26,536.995 (26,404.413)

**Table 2:** Complications by frailty divisions.

Outcome	Robust	Pre-Frail	Frail	Severely Frail
**Total Patients**	65,367 (26.0%)	76,444 (30.4%)	59,107 (23.5%)	50,679 (20.1%)
**Any Complication**	12,630 (19.3%)	18,528 (24.2%)	16,316 (27.6%)	17,679 (34.9%)
Pulmonary	8939 (13.7%)	12,699 (16.6%)	11,245 (19.0%)	11,963 (23.6%)
Thromboembolic	2181 (3.3%)	3119 (4.1%)	2558 (4.3%)	2621 (5.2%)
Renal and Genitourinary	2164 (3.3%)	3786 (5.0%)	3965 (6.7%)	5250 (10.4%)
DVT	2224 (3.4%)	3183 (4.2%)	2678 (4.5%)	2666 (5.3%)
Sepsis	711 (1.1%)	1136 (1.5%)	1200 (2.0%)	1228 (2.4%)
Infected Wound	1125 (1.7%)	1033 (1.4%)	688 (1.2%)	553 (1.1%)
Cardiac	1043 (1.6%)	1583 (2.1%)	1599 (2.7%)	2451 (4.8%)

**Table 3:** Multivariable regression model odds ratios (95% CI; *p*-value).

Outcome	mFI-5	mFI-11	CCI	Age
Died	**1.091 (1.048 to 1.135, <0.001)**	**1.088 (1.058 to 1.119, <0.001)**	**1.151 (1.140 to 1.163, <0.001)**	1.002 (0.999 to 1.006, *p* = 0.140)
Non-Home Discharge	**1.359 (1.326 to 1.394, <0.001)**	**1.330 (1.305 to 1.355, <0.001)**	**1.230 (1.219 to 1.242, <0.001)**	**1.039 (1.037 to 1.041, *p* < 0.001)**
Any Complication	**1.355 (1.323 to 1.389, <0.001)**	**1.256 (1.235 to 1.278, <0.001)**	**1.227 (1.218 to 1.237, <0.001)**	1.000 (0.998 to 1.002, *p* = 0.703)
Extended LOS	**1.147 (1.120 to 1.175, <0.001)**	**1.150 (1.131 to 1.170, <0.001)**	**1.182 (1.173 to 1.190, <0.001)**	1.001 (0.999 to 1.003, *p* = 0.338)

## Data Availability

Data are available upon reasonable request to the corresponding author after all HCUP NIS protocol training and proof of certification.

## Supplementary Materials

The following supporting information can be downloaded at https://www.mdpi.com/article/10.3390/jcm14093176/s1, Table S1: MV analysis of mFI-5; Table S2: MV Analysis of mFI-11; Table S3: MV Analysis of CCI; Table S4: MV Age Analysis.

## Abbreviations

The following abbreviations are used in this manuscript:

naSDH	Nonacute Subdural Hematoma
mFI	Modified Frailty Index
mFI-5	5-Factor Modified Frailty Index
mFI-11	11-Factor Modified Frailty Index
CCI	Charlson Comorbidity Index
LOS	Length of Stay
ROC	Receiver Operating Characteristic
AUC	Area Under the Curve
ICD	International Classification of Diseases
NIS	Nationwide Inpatient Sample
ICD-10-CM	International Classification of Diseases, Tenth Revision, Clinical Modification
ICD-10-PCS	International Classification of Diseases, Tenth Revision, Procedure Coding System
ICD-9-CM	International Classification of Diseases, Ninth Revision, Clinical Modification
OR	Odds Ratio
CI	Confidence Interval
IRB	Institutional Review Board
STROBE	Strengthening the Reporting of Observational Studies in Epidemiology
HCUP	Healthcare Cost and Utilization Project
SD	Standard Deviation
