# Supplementary material for: Impact of Frailty on Surgical Outcomes in Nonacute Subdural Hematomas: A Nationwide Analysis of 251,597 Patients over 20 Years
Source: J Clin Med. 2025 May 4;14(9):3176. doi: 10.3390/jcm14093176 (PMC12073061; doi:10.3390/jcm14093176)
Supplement: Supplementary file 1 [file jcm-14-03176-s001.zip › jcm-3590225-supplementary.pdf]

**Supplementary Table S1. MV analysis of mFI-5**

| | OR | 95% CI<br>Mortality | p-val | OR | 95% CI<br>Non-Home<br>Discharge | p-val | OR | 95% CI<br>Any<br>Complication | p-val | OR | 95% CI<br>Extended<br>LOS | p-val | GLM<br>Estimated Cost of Care | p-val |
|---|---|---|---|---|---|---|---|---|---|---|---|---|---|---|
| **mFI-5** | **1.091*** ** | **1.048 - 1.135** | **<0.0001** | **1.359*** ** | **1.326 - 1.394** | **<0.0001** | **1.355*** ** | **1.323 - 1.389** | **<0.0001** | **1.147*** ** | **1.120 - 1.175** | **<0.0001** | **2,645*** ** | **<0.0001** |
| **Sex** | | | | | | | | | | | | | | |
| Male | | Reference | | | Reference | | | Reference | | | | | Reference | |
| Female | **1.372*** ** | **1.276 - 1.476** | **<0.0001** | **1.556*** ** | **1.486 - 1.630** | **<0.0001** | **1.174*** ** | **1.121 - 1.230** | **<0.0001** | **1.227*** ** | **1.175 - 1.282** | **<0.0001** | **3,400*** ** | **<0.0001** |
| **Race** | | | | | | | | | | | | | | |
| White | | Reference | | | Reference | | | Reference | | | Reference | | Reference | |
| Black | 0.929 | 0.834 - 1.034 | 0.1770 | **1.144*** ** | **1.071 - 1.221** | **0.0001** | **1.190*** ** | **1.115 - 1.269** | **<0.0001** | **1.305*** ** | **1.227 - 1.388** | **<0.0001** | **3,442*** ** | **<0.0001** |
| Hispanic | 0.949 | 0.837 - 1.077 | 0.4180 | **0.869*** ** | **0.804 - 0.938** | **0.0004** | 1.008 | 0.931 - 1.091 | 0.8500 | 1.128*** | 1.047 - 1.215 | 0.0016 | 4,122*** | 0.0000 |
| Asian | 0.892 | 0.743 - 1.070 | 0.2180 | 0.919 | 0.821 - 1.028 | 0.1400 | 0.927 | 0.825 - 1.040 | 0.1970 | 0.992 | 0.895 - 1.098 | 0.8710 | 1,745* | 0.0532 |
| Native American | 1.022 | 0.645 - 1.618 | 0.9260 | 0.836 | 0.627 - 1.115 | 0.2230 | 1.218 | 0.911 - 1.630 | 0.1840 | 1.115 | 0.835 - 1.488 | 0.4600 | 675.9 | 0.7050 |
| Other | 0.831* | 0.676 - 1.023 | 0.0807 | 0.922 | 0.813 - 1.045 | 0.2040 | 1.048 | 0.911 - 1.206 | 0.5130 | 1.028 | 0.912 - 1.160 | 0.6490 | 2,279** | 0.0135 |
| **Primary Payer** | | | | | | | | | | | | | | |
| Privately Insured | | Reference | | | Reference | | | Reference | | | Reference | | | |
| Medicare | **1.274*** ** | **1.157 - 1.403** | **<0.0001** | **3.076*** ** | **2.919 - 3.242** | **<0.0001** | **1.122*** ** | **1.059 - 1.188** | **0.0001** | **1.196*** ** | **1.132 - 1.264** | **<0.0001** | **-3,219*** ** | **<0.0001** |
| Medicaid | **1.344*** ** | **1.152 - 1.567** | **0.0002** | **1.468*** ** | **1.344 - 1.602** | **<0.0001** | **1.457*** ** | **1.326 - 1.601** | **<0.0001** | **1.656*** ** | **1.512 - 1.813** | **<0.0001** | **7,385*** ** | **<0.0001** |
| Self Pay | **1.767*** ** | **1.456 - 2.146** | **<0.0001** | **0.661*** ** | **0.582 - 0.751** | **<0.0001** | **1.179** ** | **1.029 - 1.350** | **<0.05** | 1.006 | 0.888 - 1.139 | 0.9280 | 366.5 | 0.6680 |
| No Charge | 1.137 | 0.580 - 2.229 | 0.7090 | **0.566*** ** | **0.382 - 0.838** | **0.0045** | 1.068 | 0.731 - 1.561 | 0.7350 | **1.430** ** | **1.004 - 2.035** | **<0.05** | -252.0 | 0.8920 |
| Other | 1.072 | 0.842 - 1.364 | 0.5730 | 1.064 | 0.936 - 1.209 | 0.3430 | 0.911 | 0.784 - 1.059 | 0.2270 | 0.972 | 0.846 - 1.116 | 0.6850 | -201.7 | 0.8380 |
| **Median household income quartile for patient's ZIP Code** | **0.957** ** | **0.924 - 0.990** | **<0.05** | **0.958*** ** | **0.937 - 0.979** | **0.0001** | **0.958*** ** | **0.937 - 0.979** | **0.0001** | 0.990 | 0.969 - 1.010 | 0.3150 | **881.8*** ** | **<0.0001** |
| **Hospital Status** | | | | | | | | | | | | | | |
| Urban, Teaching | | Reference | | | Reference | | | Reference | | | Reference | | Reference | |
| Rural | 1.114 | 0.887 - 1.398 | 0.3540 | **0.801** ** | **0.676 - 0.949** | **0.0102** | **0.759*** ** | **0.649 - 0.889** | **0.0006** | **0.785*** ** | **0.674 - 0.914** | **0.0018** | **-4,382*** ** | **<0.0001** |
| Urban, Non-Teaching | 1.057 | 0.972 - 1.150 | 0.1950 | **1.259*** ** | **1.190 - 1.332** | **<0.0001** | 1.006 | 0.949 - 1.066 | 0.8420 | 0.996 | 0.941 - 1.055 | 0.9000 | **-5,404*** ** | **<0.0001** |
| **Region of the Hospital** | | | | | | | | | | | | | | |
| Northeast | | Reference | | | Reference | | | Reference | | | Reference | | Reference | |
| Midwest | 0.934 | 0.824 - 1.058 | 0.2840 | **0.751*** ** | **0.693 - 0.815** | **<0.0001** | **1.195*** ** | **1.100 - 1.297** | **<0.0001** | **0.842*** ** | **0.775 - 0.915** | **<0.0001** | -693.2 | 0.2730 |
| South | 0.974 | 0.876 - 1.083 | 0.6270 | **0.680*** ** | **0.632 - 0.730** | **<0.0001** | **1.157*** ** | **1.079 - 1.241** | **<0.0001** | 1.008 | 0.943 - 1.077 | 0.8170 | **-2,341*** ** | **<0.0001** |
| West | 0.945 | 0.842 - 1.060 | 0.3340 | **0.661*** ** | **0.610 - 0.716** | **<0.0001** | **1.206*** ** | **1.114 - 1.305** | **<0.0001** | **0.841*** ** | **0.779 - 0.907** | **<0.0001** | **7,410*** ** | **<0.0001** |

*** p<0.01, ** p<0.05, * p<0.1

**Supplementary Table S2. MV Analysis of mFI-11**

| | OR | 95% CI Mortality | p-val | OR | 95% CI Non-Home Discharge | p-val | OR | 95% CI Any Complication | p-val | OR | 95% CI Extended LOS | p-val | OR | p-val |
|---|---|---|---|---|---|---|---|---|---|---|---|---|---|---|
| **mFI-11** | 1.088*** | 1.058 - 1.119 | <0.0001 | 1.330*** | 1.305 - 1.355 | <0.0001 | 1.256*** | 1.235 - 1.278 | <0.0001 | 1.150*** | 1.131 - 1.170 | <0.0001 | 2,335*** | <0.0001 |
| **Sex** | | | | | | | | | | | | | | |
| Male | | Reference | | | Reference | | | Reference | | | Reference | | | |
| Female | 1.388*** | 1.290 - 1.493 | <0.0001 | 1.611*** | 1.538 - 1.688 | <0.0001 | 1.206*** | 1.152 - 1.264 | <0.0001 | 1.251*** | 1.197 - 1.306 | 0.0000 | 3,671*** | <0.0001 |
| **Race** | | | | | | | | | | | | | | |
| White | | Reference | | | Reference | | | Reference | | | Reference | | | |
| Black | 0.932 | 0.837 - 1.037 | 0.1960 | 1.164*** | 1.090 - 1.243 | <0.0001 | 1.220*** | 1.144 - 1.300 | <0.0001 | 1.312*** | 1.234 - 1.395 | <0.0001 | 3,607*** | <0.0001 |
| Hispanic | 0.953 | 0.840 - 1.081 | 0.4520 | 0.881*** | 0.816 - 0.952 | <0.0001 | 1.026 | 0.948 - 1.111 | 0.5200 | 1.135*** | 1.054 - 1.223 | 0.000868 | 4,251*** | <0.0001 |
| Asian | 0.895 | 0.746 - 1.074 | 0.2320 | 0.933 | 0.834 - 1.044 | 0.227 | 0.949 | 0.845 - 1.065 | 0.3730 | 0.997 | 0.900 - 1.105 | 0.956 | 1,885** | 0.0360 |
| Native American | 1.025 | 0.647 - 1.625 | 0.9150 | 0.842 | 0.631 - 1.124 | 0.244 | 1.241 | 0.928 - 1.660 | 0.1460 | 1.121 | 0.838 - 1.498 | 0.442 | 831.9 | 0.641 |
| Other | 0.833* | 0.677 - 1.024 | <0.1 | 0.925 | 0.815 - 1.049 | 0.225 | 1.060 | 0.922 - 1.219 | 0.4120 | 1.031 | 0.914 - 1.162 | 0.623 | 2,364** | 0.0103 |
| **Primary Payer** | | | | | | | | | | | | | | |
| Privately Insured | | Reference | | | Reference | | | Reference | | | Reference | | | |
| Medicare | 1.251*** | 1.135 - 1.380 | <0.0001 | 2.948*** | 2.797 - 3.108 | <0.0001 | 1.092*** | 1.031 - 1.157 | 0.0026 | 1.159*** | 1.096 - 1.226 | 1.98e-07 | -3,640*** | <0.0001 |
| Medicaid | 1.338*** | 1.148 - 1.561 | 0.0002 | 1.454*** | 1.330 - 1.588 | <0.0001 | 1.441*** | 1.312 - 1.584 | <0.0001 | 1.648*** | 1.505 - 1.804 | 0 | 7,252*** | <0.0001 |
| Self Pay | 1.779*** | 1.466 - 2.160 | 0.0000 | 0.671*** | 0.591 - 0.762 | <0.0001 | 1.186** | 1.036 - 1.357 | 0.0134 | 1.018 | 0.899 - 1.152 | 0.780 | 486.5 | 0.567 |
| No Charge | 1.149 | 0.586 - 2.253 | 0.6850 | 0.580*** | 0.391 - 0.860 | 0.00668 | 1.078 | 0.735 - 1.579 | 0.7020 | 1.458** | 1.024 - 2.077 | 0.0364 | -47.02 | 0.980 |
| Other | 1.074 | 0.844 - 1.367 | 0.5630 | 1.070 | 0.941 - 1.217 | 0.300 | 0.920 | 0.792 - 1.069 | 0.2770 | 0.974 | 0.848 - 1.119 | 0.711 | -155.8 | 0.874 |
| **Median household income quartile for patient's ZIP Code** | 0.958** | 0.925 - 0.992 | 0.0147 | 0.960*** | 0.940 - 0.982 | <0.0001 | 0.956*** | 0.935 - 0.977 | 0.0001 | 0.992 | 0.972 - 1.013 | 0.448 | 899.5*** | <0.0001 |
| **Hospital Status** | | | | | | | | | | | | | | |
| Urban, Teaching | | Reference | | | Reference | | | Reference | | | Reference | | Reference | |
| Rural | 1.119 | 0.891 - 1.406 | 0.3340 | 0.810** | 0.683 - 0.961 | <0.05 | 0.762*** | 0.651 - 0.892 | 0.0007 | 0.792*** | 0.679 - 0.924 | 0.00298 | -4,283*** | <0.0001 |
| Urban, Non-Teaching | 1.059 | 0.973 - 1.152 | 0.1830 | 1.267*** | 1.197 - 1.341 | <0.0001 | 1.005 | 0.948 - 1.066 | 0.8590 | 0.999 | 0.944 - 1.058 | 0.979 | -5,387*** | <0.0001 |
| **Region of the Hospital** | | | | | | | | | | | | | | |
| Northeast | | Reference | | | Reference | | | Reference | | | Reference | | Reference | |
| Midwest | 0.926 | 0.817 - 1.049 | 0.2280 | 0.734*** | 0.677 - 0.797 | <0.0001 | 1.179*** | 1.086 - 1.280 | 0.0001 | 0.829*** | 0.763 - 0.901 | 1.01e-05 | -877.0 | 0.162 |
| South | 0.972 | 0.874 - 1.080 | 0.5950 | 0.675*** | 0.628 - 0.725 | <0.0001 | 1.157*** | 1.079 - 1.241 | <0.0001 | 1.004 | 0.939 - 1.073 | 0.904 | -2,392*** | <0.0001 |
| West | 0.943 | 0.841 - 1.058 | 0.3190 | 0.657*** | 0.606 - 0.712 | <0.0001 | 1.206*** | 1.114 - 1.305 | <0.0001 | 0.838*** | 0.776 - 0.904 | 4.70e-06 | 7,421*** | <0.0001 |

Standard errors in parentheses

*** p<0.01, ** p<0.05, * p<0.1

**Supplementary Table S3. MV Analysis of CCI**

| | **OR** | **95% CI** Mortality | **p-val** | **OR** | **95% CI** Non-Home Discharge | **p-val** | **OR** | **95% CI** Any Complication | **p-val** | **OR** | **95% CI** Extended LOS | **p-val** | **GLM** Estimated Cost of Care | p-val |
|---|---|---|---|---|---|---|---|---|---|---|---|---|---|---|
| **CCI** | **1.151*** ** | **1.140 - 1.163** | **<0.0001** | **1.230*** ** | **1.219 - 1.242** | **<0.0001** | **1.227*** ** | **1.218 - 1.237** | **<0.0001** | **1.182*** ** | **1.173 - 1.190** | **<0.0001** | **2,494*** ** | **<0.0001** |
| **Sex** | | | | | | | | | | | | | | |
| Male | | Reference | | | Reference | | | Reference | | | Reference | | Reference | |
| Female | **1.374*** ** | **1.277 - 1.479** | **<0.0001** | **1.563*** ** | **1.491 - 1.639** | **<0.0001** | **1.168*** ** | **1.113 - 1.225** | **<0.0001** | **1.232*** ** | **1.178 - 1.288** | **<0.0001** | **3,198*** ** | **<0.0001** |
| **Race** | | | | | | | | | | | | | | |
| White | | Reference | | | Reference | | | Reference | | | Reference | | Reference | |
| Black | **0.880** ** | **0.790 - 0.982** | **<0.05** | **1.164*** ** | **1.088 - 1.245** | **<0.0001** | **1.178*** ** | **1.102 - 1.260** | **<0.0001** | **1.274*** ** | **1.196 - 1.359** | **<0.0001** | **3,089*** ** | **<0.0001** |
| Hispanic | 0.941 | 0.828 - 1.070 | 0.3520 | **0.893*** ** | **0.825 - 0.967** | **<0.01** | 1.024 | 0.942 - 1.113 | 0.5780 | **1.139*** ** | **1.054 - 1.230** | **0.0009** | **4,097*** ** | **<0.0001** |
| Asian | 0.907 | 0.756 - 1.088 | 0.2940 | 0.987 | 0.884 - 1.103 | 0.8190 | 0.989 | 0.878 - 1.114 | 0.8510 | 1.028 | 0.926 - 1.140 | 0.6080 | **2,364*** ** | **0.0053** |
| Native American | 1.028 | 0.648 - 1.631 | 0.9060 | 0.832 | 0.619 - 1.119 | 0.2240 | 1.259 | 0.937 - 1.690 | 0.1260 | 1.126 | 0.838 - 1.515 | 0.4310 | 789.6 | 0.6490 |
| Other | **0.830*** | **0.674 - 1.022** | **<0.1** | 0.955 | 0.842 - 1.084 | 0.4750 | 1.080 | 0.939 - 1.242 | 0.2820 | 1.042 | 0.923 - 1.176 | 0.5090 | **2,519*** ** | **<0.01** |
| **Primary Payer** | | | | | | | | | | | | | | |
| Privately Insured | | Reference | | | Reference | | | Reference | | | Reference | | Reference | |
| Medicare | **1.156*** ** | **1.049 - 1.274** | **<0.01** | **2.992*** ** | **2.837 - 3.156** | **<0.0001** | 1.033 | 0.974 - 1.096 | 0.2820 | **1.079*** ** | **1.020 - 1.143** | **0.0085** | **-4,607*** ** | **<0.0001** |
| Medicaid | **1.289*** ** | **1.102 - 1.506** | **<0.01** | **1.407*** ** | **1.283 - 1.543** | **<0.0001** | **1.402*** ** | **1.271 - 1.547** | **<0.0001** | **1.620*** ** | **1.474 - 1.779** | **0.0000** | **6,682*** ** | **<0.0001** |
| Self Pay | **1.930*** ** | **1.589 - 2.344** | **<0.0001** | **0.683*** ** | **0.600 - 0.777** | **<0.0001** | **1.280*** ** | **1.115 - 1.470** | **0.0005** | 1.086 | 0.958 - 1.231 | 0.1970 | **1,394*** | **<0.1** |
| No Charge | 1.365 | 0.691 - 2.696 | 0.3700 | 0.632** | 0.427 - 0.936 | 0.0218 | 1.271 | 0.865 - 1.867 | 0.2220 | **1.700*** ** | **1.189 - 2.431** | **0.0036** | 2,185 | 0.2390 |
| Other | 1.099 | 0.860 - 1.404 | 0.4520 | 1.111 | 0.976 - 1.264 | 0.1120 | 0.952 | 0.815 - 1.112 | 0.5340 | 1.002 | 0.869 - 1.154 | 0.9830 | 287.3 | 0.7600 |
| **Median household income quartile for patient's ZIP Code** | **0.956** ** | **0.923 - 0.990** | **<0.05** | **0.943*** ** | **0.922 - 0.964** | **<0.0001** | **0.941*** ** | **0.920 - 0.963** | **<0.0001** | 0.986 | 0.965 - 1.008 | 0.2030 | **801.7*** ** | **<0.0001** |
| **Hospital Status** | | | | | | | | | | | | | | |
| Urban, Teaching | | Reference | | | Reference | | | Reference | | | Reference | | Reference | |
| Rural | 1.210 | 0.960 - 1.524 | 0.1060 | **0.827** ** | **0.698 - 0.980** | **<0.05** | **0.811*** ** | **0.694 - 0.947** | **<0.01** | **0.837** ** | **0.719 - 0.973** | **<0.05** | **-3,389*** ** | **<0.0001** |
| Urban, Non-Teaching | **1.103** ** | **1.013 - 1.201** | **<0.05** | **1.303*** ** | **1.230 - 1.379** | **<0.0001** | 1.045 | 0.985 - 1.109 | 0.1430 | 1.033 | 0.974 - 1.096 | 0.2810 | **-4,803*** ** | **<0.0001** |
| **Region of the Hospital** | | | | | | | | | | | | | | |
| Northeast | | Reference | | | Reference | | | Reference | | | Reference | | Reference | |
| Midwest | **0.855** ** | **0.753 - 0.971** | **<0.05** | **0.696*** ** | **0.640 - 0.758** | **<0.0001** | **1.104** ** | **1.014 - 1.201** | **0.0225** | **0.765*** ** | **0.702 - 0.834** | **<0.0001** | **-1,902*** ** | **<0.01** |
| South | 0.927 | 0.833 - 1.031 | 0.1640 | **0.644*** ** | **0.598 - 0.694** | **<0.0001** | **1.110*** ** | **1.032 - 1.194** | **0.0053** | 0.962 | 0.898 - 1.031 | 0.2720 | **-3,065*** ** | **<0.0001** |
| West | **0.882** ** | **0.786 - 0.991** | **0.0347** | **0.610*** ** | **0.562 - 0.663** | **<0.0001** | **1.125*** ** | **1.037 - 1.221** | **0.0048** | **0.777*** ** | **0.718 - 0.840** | **<0.0001** | **6,429*** ** | **<0.0001** |

Standard errors in parentheses

*** p<0.01, ** p<0.05, * p<0.1

**Supplementary Table S4. MV Age Analysis**

| | OR | 95% CI | p-val | OR | 95% CI | p-val | OR | 95% CI | p-val | OR | 95% CI | p-val | GLM | p-val |
|---|---|---|---|---|---|---|---|---|---|---|---|---|---|---|
| | | Mortality | | | Non-Home Discharge | | | Any Complication | | | Extended LOS | | Estimated Cost of Care | |
| **Age** | 1.002 | 0.999 - 1.006 | 0.1400 | **1.039*** ** | **1.037 - 1.041** | **<0.0001** | 1.000 | 0.998 - 1.002 | 0.7030 | 1.001 | 0.999 - 1.003 | 0.338 | **-214.3*** ** | **<0.0001** |
| **Sex** | | | | | | | | | | | | | | |
| Male | | Reference | | | Reference | | | Reference | | | | | Reference | |
| Female | **1.369*** ** | **1.273 - 1.472** | **<0.0001** | **1.603*** ** | **1.529 - 1.681** | **<0.0001** | **1.155*** ** | **1.104 - 1.209** | **<0.0001** | **1.220*** ** | **1.168 - 1.274** | **<0.0001** | **3,144*** ** | **<0.0001** |
| **Race** | | | | | | | | | | | | | | |
| White | | Reference | | | Reference | | | Reference | | | | | Reference | |
| Black | 0.952 | 0.855 - 1.060 | 0.3710 | **1.359*** ** | **1.272 - 1.452** | **<0.0001** | 1.263*** | 1.184 - 1.347 | 0.0000 | **1.344*** ** | **1.264 - 1.429** | **<0.0001** | **3,464*** ** | **<0.0001** |
| Hispanic | 0.962 | 0.848 - 1.091 | 0.5450 | 0.956 | 0.884 - 1.034 | 0.2620 | 1.039 | 0.960 - 1.125 | 0.3430 | **1.146*** ** | **1.064 - 1.234** | **0.0003** | **4,060*** ** | **<0.0001** |
| Asian | 0.904 | 0.753 - 1.084 | 0.2750 | 0.926 | 0.827 - 1.037 | 0.1840 | 0.981 | 0.874 - 1.100 | 0.7400 | 1.015 | 0.917 - 1.125 | 0.769 | **2,385*** ** | **0.0083** |
| Native American | 1.039 | 0.655 - 1.648 | 0.8710 | 0.962 | 0.715 - 1.293 | 0.7950 | 1.254 | 0.940 - 1.673 | 0.1240 | 1.132 | 0.849 - 1.510 | 0.397 | 292.9 | 0.8660 |
| Other | 0.841 | 0.683 - 1.035 | 0.1020 | 0.983 | 0.861 - 1.122 | 0.8010 | 1.080 | 0.939 - 1.243 | 0.2810 | 1.043 | 0.925 - 1.176 | 0.487 | **2,207** ** | **0.0176** |
| **Primary Payer** | | | | | | | | | | | | | | |
| Privately Insured | | Reference | | | Reference | | | Reference | | | | | Reference | |
| Medicare | **1.256*** ** | **1.123 - 1.405** | **0.0001** | **1.807*** ** | **1.701 - 1.919** | **<0.0001** | **1.244*** ** | **1.166 - 1.328** | **<0.0001** | **1.230*** ** | **1.155 - 1.309** | **<0.0001** | **1,317*** ** | **0.0017** |
| Medicaid | **1.358*** ** | **1.163 - 1.585** | **0.0001** | **1.788*** ** | **1.630 - 1.962** | **<0.0001** | **1.443*** ** | **1.313 - 1.585** | **<0.0001** | **1.657*** ** | **1.512 - 1.815** | **<0.0001** | **6,344*** ** | **<0.0001** |
| Self Pay | **1.759*** ** | **1.448 - 2.137** | **<0.0001** | **0.739*** ** | **0.649 - 0.842** | **<0.0001** | 1.110 | 0.968 - 1.274 | 0.1350 | 0.984 | 0.869 - 1.115 | 0.803 | -1,108 | 0.2010 |
| No Charge | 1.116 | 0.569 - 2.189 | 0.7490 | **0.583*** ** | **0.391 - 0.869** | **0.0080** | 0.970 | 0.663 - 1.419 | 0.8740 | 1.374* | 0.968 - 1.950 | 0.0754 | -1,962 | 0.3120 |
| Other | 1.073 | 0.843 - 1.366 | 0.5660 | 1.023 | 0.900 - 1.162 | 0.7310 | 0.929 | 0.800 - 1.079 | 0.3340 | 0.978 | 0.851 - 1.124 | 0.753 | 241.7 | 0.8080 |
| **Median household income quartile for patient's ZIP Code** | **0.950*** ** | **0.918 - 0.983** | **0.0033** | **0.925*** ** | **0.905 - 0.945** | **0.0000** | **0.938*** ** | **0.917 - 0.959** | **<0.0001** | **0.979** ** | **0.960 - 1.000** | **0.0473** | **851.0*** ** | **0.0000** |
| **Hospital Status** | | | | | | | | | | | | | | |
| Urban, Teaching | | Reference | | | Reference | | | Reference | | | | | Reference | |
| Rural | 1.098 | 0.877 - 1.375 | 0.4160 | **0.737*** ** | **0.624 - 0.870** | **0.0003** | **0.733*** ** | **0.631 - 0.852** | **0.0001** | **0.770*** ** | **0.664 - 0.892** | **0.0005** | **-4,527*** ** | **<0.0001** |
| Urban, Non-Teaching | 1.048 | 0.963 - 1.139 | 0.2790 | **1.178*** ** | **1.113 - 1.247** | **<0.0001** | 0.986 | 0.930 - 1.046 | 0.6490 | 0.986 | 0.931 - 1.044 | 0.626 | **-5,147*** ** | **<0.0001** |
| **Region of the Hospital** | | | | | | | | | | | | | | |
| Northeast | | Reference | | | Reference | | | Reference | | | | | Reference | |
| Midwest | 0.946 | 0.835 - 1.072 | 0.3850 | **0.792*** ** | **0.727 - 0.863** | **<0.0001** | **1.242*** ** | **1.142 - 1.351** | **<0.0001** | **0.858*** ** | **0.790 - 0.932** | **0.0003** | -511.8 | 0.4180 |
| South | 0.982 | 0.884 - 1.091 | 0.7400 | **0.704*** ** | **0.654 - 0.758** | **<0.0001** | **1.183*** ** | **1.103 - 1.269** | **<0.0001** | 1.019 | 0.954 - 1.089 | 0.578 | **-2,295*** ** | **<0.0001** |
| West | 0.952 | 0.848 - 1.068 | 0.3990 | **0.680*** ** | **0.626 - 0.739** | **<0.0001** | **1.227*** ** | **1.133 - 1.329** | **<0.0001** | **0.849*** ** | **0.787 - 0.916** | **<0.0001** | **7,271*** ** | **<0.0001** |

*** p<0.01, ** p<0.05, * p<0.1
